# Discovery and Genetic Characterization of Novel Paramyxoviruses Related to the Genus *Henipavirus* in *Crocidura* Species in the Republic of Korea

**DOI:** 10.3390/v13102020

**Published:** 2021-10-07

**Authors:** Seung-Ho Lee, Kijin Kim, Jongwoo Kim, Jin Sun No, Kyungmin Park, Shailesh Budhathoki, Seung Ho Lee, Jingyeong Lee, Seung Hye Cho, Seungchan Cho, Geum-Young Lee, Jusun Hwang, Heung-Chul Kim, Terry A. Klein, Chang-Sub Uhm, Won-Keun Kim, Jin-Won Song

**Affiliations:** 1 Department of Microbiology, Korea University College of Medicine, Seoul 02841, Korea; leeds1104@korea.ac.kr (S.-H.L.); hotdog442@korea.ac.kr (J.K.); kmpark0131@korea.ac.kr (K.P.); meales@korea.ac.kr (S.H.L.); yoj0702@korea.ac.kr (J.L.); schanchan@korea.ac.kr (S.C.); gemyeng002@korea.ac.kr (G.-Y.L.); 2 Saarland University Saarbrücken Campus, 66123 Saarbrücken, Germany; skkujin@gmail.com; 3 BK21 Graduate Program, Department of Biomedical Sciences, Korea University College of Medicine, Seoul 02841, Korea; 4 Division of High-Risk Pathogens, Bureau of Infectious Diseases Diagnosis Control, Korea Disease Control and Prevention Agency, Cheongju 28159, Korea; njs2564@gmail.com; 5 Department of Microbiology, College of Medicine, Hallym University, Chuncheon 24252, Korea; shailesh.sas24@gmail.com; 6 Department of Biomedical Science, College of Natural Sciences, Hallym University, Chuncheon 24252, Korea; hoahae@naver.com; 7 Wildlife Conservation Society, Health Program, Bronx, NY 10460, USA; jusunhwang@gmail.com; 8 Force Health Protection and Preventive Medicine, Medical Department Activity-Korea/65th Medical Brigade, Unit 15281, APO AP 96271-5281, USA; hungchol.kim2.ln@mail.mil (H.-C.K.); terry.a.klein2.civ@mail.mil (T.A.K.); 9 Department of Anatomy, Korea University College of Medicine, Seoul 02841, Korea; uhmcs@korea.ac.kr; 10 Institute of Medical Science, College of Medicine, Hallym University, Chuncheon 24252, Korea

**Keywords:** *Crocidura* paramyxovirus, novel virus discovery, next-generation sequencing, genetic characterization and diversity, potential zoonosis

## Abstract

Paramyxoviruses, negative-sense single-stranded RNA viruses, pose a critical threat to human public health. Currently, 78 species, 17 genera, and 4 subfamilies of paramyxoviruses are harbored by multiple natural reservoirs, including rodents, bats, birds, reptiles, and fish. Henipaviruses are critical zoonotic pathogens that cause severe acute respiratory distress and neurological diseases in humans. Using reverse transcription-polymerase chain reaction, 115 *Crocidura* species individuals were examined for the prevalence of paramyxovirus infections. Paramyxovirus RNA was observed in 26 (22.6%) shrews collected at five trapping sites, Republic of Korea. Herein, we report two genetically distinct novel paramyxoviruses (genus: *Henipavirus*): Gamak virus (GAKV) and Daeryong virus (DARV) isolated from *C. lasiura* and *C. shantungensis*, respectively. Two GAKVs and one DARV were nearly completely sequenced using next-generation sequencing. GAKV and DARV contain six genes (3′-N-P-M-F-G-L-5′) with genome sizes of 18,460 nucleotides and 19,471 nucleotides, respectively. The phylogenetic inference demonstrated that GAKV and DARV form independent genetic lineages of *Henipavirus* in *Crocidura* species. GAKV-infected human lung epithelial cells elicited the induction of type I/III interferons, interferon-stimulated genes, and proinflammatory cytokines. In conclusion, this study contributes further understandings of the molecular prevalence, genetic characteristics and diversity, and zoonotic potential of novel paramyxoviruses in shrews.

## 1. Introduction

Emerging zoonotic diseases are a critical cause of recent virus outbreaks, public health threats, and socio-economic burdens [1,2,3]. Severe acute respiratory syndrome coronavirus 2 occurred recently due to virus spillover or transmission, particularly in situations that promote frequent contact among wild animals, livestock, and humans [4]. In South America in 2015 and 2016, the Zika virus (ZIKV) epidemics are of medical significance since the virus presents severe complications, including Zika fever, congenital brain abnormalities, or neurological symptoms [5,6]. Bat- and rodent-borne zoonoses, such as Ebola virus, Lassa fever virus, and Hantavirus, impact a wide range of environmental, biological, ecological, and socio-economic drivers in vulnerability to the diseases [7,8]. Furthermore, there are no effective therapeutics and vaccines against unknown infectious viruses. Thus, continuous surveillance and characterization of potential zoonotic viruses from wildlife species are required to ensure preparedness and responsiveness against new emerging virus outbreaks.

Shrews, belonging to the family *Soricidae* (Mammalia: Eulipotyphla), are one of the largest and most abundant mammalian groups worldwide [9]. *Crocidura* species are natural reservoir hosts that carry pathogens that are detrimental to humans [10]. These species are widely distributed in various habitats, such as rural areas, agricultural fields, and forests. Recently, potential cases of shrew-transmitted disease, caused by Borna disease virus 1, have been identified in brain tissue from patients with encephalitis [10]. However, how the causative virus is transmitted from shrews to humans remains unknown. Additionally, shrews harbor several zoonotic viruses, including arenaviruses [11], coronaviruses [12], hantaviruses [13,14,15,16,17], rotaviruses [18], and hepadnaviruses [19]. Metagenomic studies and continuous surveillance of potential viruses in small mammals provide clues for preventive and mitigating strategies against new emerging and re-emerging infectious diseases [20,21,22,23,24,25].

Paramyxovirus is a non-segmented, negative-sense, single-stranded RNA virus. *Orthoparamyxovirinae*, the largest subfamily, is classified into nine genera and comprises 34 recognized species [26]. Paramyxoviruses have various host ranges, including mammals, birds, reptiles, and fish [27]. In mammals, bats possess a variety of paramyxoviruses (genus: *Henipavirus*, *Narmovirus*, *Respirovirus*, and *Rubulavirus*) worldwide [28]. Recently, paramyxoviruses have been found in multiple natural hosts, such as rodents [29,30,31] and cats [32]. Different types of paramyxoviruses have been discovered and isolated in the Republic of Korea (ROK) (genus: *Orthoavulavirus*, *Shaanvirus*, and *Jeilongvirus*). Avian avulaviruses have been isolated from wild migratory birds [33]. The bat paramyxovirus (genus: *Shaanvirus*) was discovered in fecal matter from the common bent-wing bat, eastern long-fingered bat, and eastern water bat [34,35]. The striped field mouse was found to harbor Paju Apodemus paramyxovirus 1 and 2 (PAPV-1 and -2) in kidney tissues [30].

Henipaviruses are critical zoonotic pathogens that cause severe acute respiratory distress and neurological diseases in humans [27,36,37]. The emergence of the Nipah virus (NiV) has led to high morbidity and mortality [38,39]. The genus *Henipavirus* consists of five species, including Hendra, Nipah, Mòjiāng, Cedar, and Kumasi henipaviruses (‘ICTV Master Species List 2018.v1′ 2019). *Henipavirus* has been mainly found in bats, but it was recently found in rats in China and shrews in Zambia [40,41]. In 2012, a unique rodent-borne henipavirus, Mòjiāng virus (MojV), was discovered by an epidemiological investigation of lethal pneumonialike illness cases in a mineshaft in Mojiang, China [41]. Although MojV is only identified by sequence data, the deficiency of a conserved ephrin receptor-binding domain in MojV attachment glycoprotein suggested a differential viral entry mechanism independently of well-known henipavirus receptors, including A- and B-class ephrins, sialic acid, and CD150 [42,43]. The epidemiology, genetic diversity, and potential zoonosis of MojV remain to be unknown owing to the lack of isolation.

In this study, 115 individuals belonging to species of *Crocidura* (*C. lasiura* and *C. shantungensis*) were examined for the geographical spread, genomic characterization, and phylogenetic relationship of novel shrew-borne henipaviruses, Gamak virus (GAKV) and Daeryong virus (DARV). These paramyxoviruses were given the names ‘Gamak virus’ and ‘Daeryong virus’ from the first discovered location of origin (Mount Gamak and Daeryong, respectively). GAKV, DARV, and rodent-borne henipavirus (MojV) appear to be distinct from previously identified bat-borne henipaviruses (Hendra virus, NiV, Cedar virus, and Kumasi virus), suggesting that they may be novel species within the genus *Henipavirus*. Infection with GAKV elicited the expression of type I/III interferons (IFNs), IFN-stimulated genes (ISGs), and proinflammatory cytokines in A549 cells. Therefore, this report demonstrates the molecular prevalence, genetic characteristics and diversity, and virus–host interactions of paramyxoviruses in shrews, ROK.

## 2. Materials and Methods

### 2.1. Ethics Statement

The US Forces Korea (USFK) approved the animal trapping procedure by USFK Regulation 40–1 “Prevention, Surveillance, and Treatment of Hemorrhagic Fever with Renal Syndrome”. The trapping, experiments, and handling of animals were conducted under a protocol approved by the Korea University Institutional Animal Care and Use Committee (KUIACUC, #2016–0049). 

### 2.2. Animal Capture and RNA Extraction

Small mammals were captured during 2017–2018 using Sherman traps (8 cm × 9 cm × 23 cm; H. B. Sherman, Tallahassee, FL, USA). A total of 115 individuals belonging to *Crocidura* species were collected from the previous study [30]. Shrews were identified using morphological criteria and polymerase chain reaction (PCR), when required. Serum, brain, lung, spleen, kidney, and liver tissues were collected aseptically and frozen at −80 °C until use. Total RNA was extracted from the kidney tissues of shrews using TRI Reagent Solution (AMBION Inc., Austin, TX, USA) according to the manufacturer’s instructions.

### 2.3. Metagenomic Next-Generation Sequencing (NGS) Using Sequence-Independent, Single-Primer Amplification (SISPA)

cDNA was generated from the RNA extracted from shrew-borne paramyxovirus-infected cells and kidney tissues using SISPA method was carried out according to a protocol described previously [30].

### 2.4. NGS for Illumina MiSeq

The cDNA library was prepared using the TruSeq Nano DNA LT Sample Preparation Kit (Illumina, San Diego, CA, USA), and the amplicon was size-selected, A-tailed, ligated with indexes and adaptors, and enriched. The library was verified for quality and size with Agilent 2100 Bioanalyzer (Agilent High Sensitivity DNA kit, Agilent Technologies) and sequenced with MiSeq reagent V2 (Illumina) using MiSeq benchtop sequencer (Illumina) with 2 × 150 bp. 

### 2.5. NGS for Illumina HiSeq

Libraries were prepared by using the NEBNext Ultra II Directional RNA-Seq Kit (New England Biolabs, Ipswich, MA, USA). Additionally, mRNA was isolated using the Poly(A) RNA Selection Kit (Lexogen, Vienna, Austria). The mRNAs were synthesized for cDNA and sheared according to the manufacturer’s instructions. The fragments were size-selected, A-tailed, ligated with indexes and adaptors, and enriched by PCR. The libraries were evaluated for the mean fragment size by the Agilent 2100 bioanalyzer (DNA High Sensitivity Kit). Quantification was performed by using a library quantification kit and StepOne Real-Time PCR System (Life Technologies). Libraries were conducted as paired-end 100 sequencing by the HiSeq X10 system (Illumina).

### 2.6. Extraction of Paramyxoviral Genome Sequences from NGS Data

The reads were trimmed with Trimmomatic (v.0.36) to remove adapter sequences for the metagenomic approach [44]. Aligned reads against the host genomic sequences were excluded using Bowtie2 (v.2.2.6) [45]. The complete mitochondrial sequence of the species on the National Center for Biotechnology Information (NCBI) RefSeq was utilized as a host reference [46]. The remaining reads were filtered for quality by FaQCs (v0.11.5), and *de novo* assembly was conducted by using SPAdes (v3.11.1) [47,48]. The assembled contigs were subsequently analyzed in the NCBI RefSeq database consisting of complete viral genomes (updated in June 2019) by nucleotide-Basic Local Alignment Search Tool (BLASTn) (v2.6.0). In addition, for the reference mapping strategy, adaptor and index sequences of reads were trimmed, and low-quality sequences were filtered using the CLC Genomics Workbench v.7.5.2 (CLC Bio, Cambridge, MA, USA). The genome sequences of Hendra virus (HeV), NiV, and MojV were used in the reference mapping method. The consensus genomic sequences of GAKV and DARV were determined by combining viral contigs extracted from CLC analysis and *de novo* assembly.

### 2.7. Reverse Transcription-Polymerase Chain Reaction (RT-PCR) Screening of Samples from Crocidura Species Individuals for Paramyxovirus

RNA was used for cDNA synthesis using a high-capacity RNA-to-cDNA kit (Applied Biosystems, Foster City, CA, USA). First and nested PCRs were conducted in a 25 μL reaction mixture containing 2.5 U of Ex Taq DNA polymerase (TaKaRa BIO Inc., Shiga, Japan), 1.5 μg of cDNA, and 10 pM of pan-paramyxoviral primers [49]. PCR condition and purification were carried out according to a protocol described previously [30]. Sequencing was performed in both directions of each PCR product using a BigDye Terminator v3.1 Cycle Sequencing Kit (Applied Biosystems) on an automated sequencer (ABI 3730XL DNA Analyzer; Applied Biosystems).

### 2.8. Rapid Amplification of cDNA Ends (RACE) PCR

The 3′ and 5′ terminal genome sequences of paramyxovirus were determined by using a SMARTer^®^ RACE 5′/3′ Kit (Takara Bio), according to the manufacturer’s specifications. PCR products were purified by the LaboPass PCR Purification Kit (Cosmo Genetech). Sequencing was conducted in both directions of each PCR product using the BigDye Terminator v3.1 Cycle Sequencing Kit (Applied Biosystems) on an automated sequencer (Applied Biosystems).

### 2.9. Mitochondrial DNA (mtDNA) Analysis

DNA was extracted from liver or lung tissues using a High Pure PCR template preparation kit (Roche, Basel, Switzerland). The *cytochrome b* gene of mtDNA was identified by universal primers [50].

### 2.10. Phylogenetic Analysis

The viral genomic sequences were aligned with MUSCLE algorithm in MEGA v.7.0 [51] and trimmed using the Lasergene program v.5 (DNASTAR, Madison, WI, USA). Phylogenetic analyses were conducted by the maximum likelihood method according to the best-fit substitution model (GTR+G+I) using MEGA 7.0. Support for the topologies was evaluated by bootstrapping for 1000 iterations. Additionally, the Markov chain Monte Carlo (MCMC) method and the BEAST package (v.1.10.4) as the Bayesian inference method were implemented to infer Bayesian phylogenetic trees [52]. The MCMC chain length was established to 100 million states by sampling every 50,000 states. The results of parameters produced sufficient sample sizes (ESS > 200). Maximum clade credibility trees were generated using TreeAnnotator (v.1.10.4) and FigTree (v.1.4.0).

### 2.11. Virus–Host Co-Divergence Analysis

To compare different phylogenetic links matching between paramyxoviruses and their hosts, a tanglegram algorithm was performed. The auxiliary lines in the center connect the phylogenetic trees. The complexity between dendrograms of phylogenies was diminished to a maximum before complete reconciliation analysis. The method was implemented using the R package “dendextend” [53].

### 2.12. Cell Lines

Vero E6 cells (ATCC, #DR-L2785) and human lung adenocarcinoma cells (A549) (ATCC, #CCL-185) were obtained from the American Type Culture Collection (ATCC, Manassas, VA, USA). Vero E6 and A549 cells were grown in Dulbecco’s modified Eagle’s medium (DMEM) supplemented with 10% fetal bovine serum (FBS), 1 mM sodium pyruvate, 2 mL of L-glutamine, and 50 mg/mL gentamicin at 37 °C in a 5% carbon dioxide (CO_2_) incubator.

### 2.13. Virus Isolation

Tissue homogenates of kidneys from GAKV-positive samples were inoculated on Vero E6 cells. The inoculum was removed after one and a half hours of adsorption. The culture was maintained with 5.5 mL of DMEM containing 5% FBS at 37 °C in a 5% CO_2_ incubator. Cytopathic effects were inspected daily using an inverted microscope.

### 2.14. Plaque Assay

Vero E6 cells were prepared at a density of 1.5 × 10^6^ cells per well onto 6-well plates. The cells were washed twice with phosphate-buffered saline (PBS) and inoculated serially with 10-fold diluted viruses. A 1:1 medium-melting-point agarose and overlay medium mix was added on the cells after 90 m of incubation at 37 °C with constant shaking. The agarose overlay was discarded at day 6. The plaques were stained with 0.1% crystal violet in 10% formaldehyde for visualization.

### 2.15. Electron Microscopy

GAKV-inoculated Vero E6 cells were collected at 5 d postinfection and fixed with 2% paraformaldehyde and 2.5% glutaraldehyde in 0.1 M PBS (pH 7.4). Ultrathin sections were placed on 400-mesh square copper electron microscopy grids (Electron Microscopy Sciences) and observed by using a transmission electron microscope (Model H-7650; Hitachi, Japan).

### 2.16. In Vitro Infection

A total of 1 × 10^6^ cells were infected with GAKV at a multiplicity of infection of 0.02. The samples were then collected at 2, 6, 12, 18, and 24 h postinfection (hpi). The mRNA genes of type I/III IFNs, ISGs, and proinflammatory cytokines were examined by using the primers (Table S1).

### 2.17. Statistical Analysis

Statistical analyses were conducted as shown in figures using the GraphPad Prism v.5.00 for Windows (GraphPad Software, San Diego, CA, USA; www.graphpad.com (accessed on 1 September 2021)).

## 3. Results

### 3.1. Discovery and Epidemiological Prevalence of Novel Henipaviruses in Shrews

**Figure 1:**
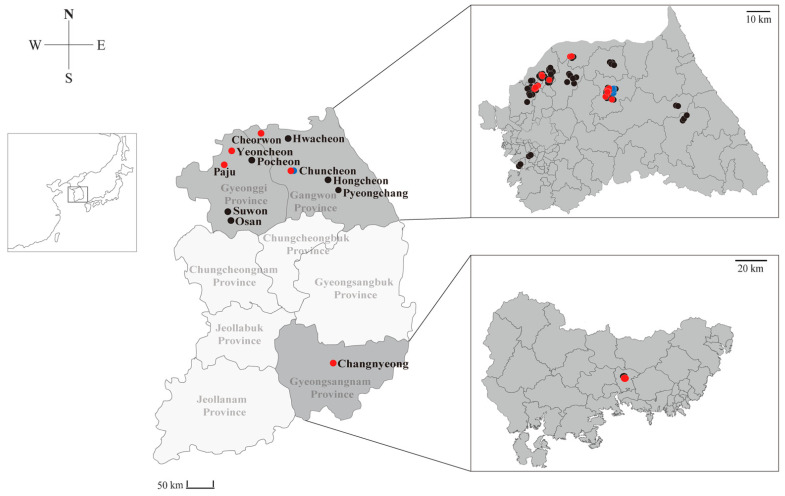
Geographic locations of the Republic of Korea, showing the shrew trapping sites described in this study. The trapping sites include Cheorwon, Chuncheon, Hwacheon, Hongcheon, and Pyeongchang in Gangwon Province; Osan, Paju, Pocheon, Suwon, and Yeoncheon in Gyeonggi Province; and Changnyeong in Gyeongsangnam Province. Red and blue dots represent trapping sites for shrews positive for the presence of Gamak virus (GAKV) and Daeryong virus (DARV), respectively. Black dots indicate that trapping sites where paramyxoviral RNA was not detected in the shrews. This figure was constructed by using Adobe Illustrator CS6 (http://www.adobe.com/products/illustrator.html, 3 March 2021).

A total of 115 shrews (94 *C. lasiura* and 21 *C. shantungensis*) were captured in 11 regions of the ROK from 2017 to 2018. All samples were obtained from Gangwon (Cheorwon, Chuncheon, Hwacheon, Hongcheon, and Pyeongchang), Gyeonggi (Osan, Paju, Pocheon, Suwon, and Yeoncheon), and Gyeongsangnam (Changnyeong) Provinces (Figure 1). To detect paramyxovirus RNA, we performed RT-PCR using consensus primers targeting the partial sequence of L gene of paramyxoviruses. In total, 20 (21.3%) *C. lasiura* and six (28.6%) *C. shantungensis* were positive for Gamak and Daeryong henipaviruses, respectively (Table 1 and Table S2). GAKV was discovered in animals from five collection sites (Paju, Yeoncheon in Gyeonggi Province, Cheorwon, Chuncheon in Gangwon Province, and Changnyeong in Gyeongsangnam Province), whereas DARV was detected in animals from one collection site (Chuncheon in Gangwon Province) (Tables S3 and S4). The geographic prevalence of GAKV followed 11/40 (27.5%) in Gangwon Province, 9/52 (17.3%) in Gyeonggi Province, and 1/2 (50.0%) in Gyeongsangnam Province, while DARV was detected only in Gangwon Province. A total of 16 of 58 (27.6%) males and 5 of 36 (13.9%) females were detected for GAKV RNA. Subadult (˂10.0 g) and adult (10–20 g) *C. lasiura* harbored GAKV infection with 11/54 (20.4%) and 10/40 (25.0%), respectively. GAKV-positive shrews were found in spring, summer, and autumn. A total of one of nine males (11.1%) and four of 12 females (33.3%) infected DARV. Three subadult *C. shatungensis* (˂5.0 g) were positive for DARV, whereas two adult *C. shatungensis* (≥5.0 g) were infected with the virus. DARV-infected shrews were found only in autumn. 

### 3.2. Isolation of Novel Henipaviruses in Shrews

**Figure 2:**
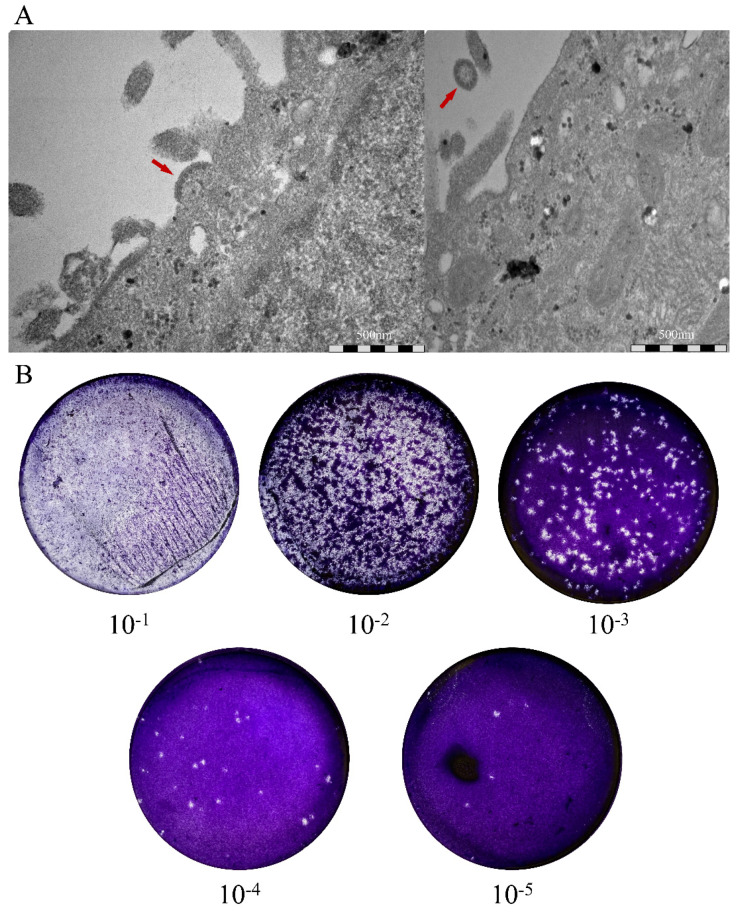
Transmission electron micrograph and quantitation of Gamak virus (GAKV). (**A**) The appearance of GAKV isolate was obtained by using the transmission electron microscopy. Red arrows indicate virus particles. (**B**) The plaque assay shows infectious GAKV inoculated onto Vero E6 cells. Each plate well represents the quantitation of infectious particles at serial dilutions from 1:10 to 1:10^5^ of the virus stock.

GAKV was isolated from the kidney tissues of Cl17-32 using a cell culture-based method. The first isolate of GAKV was confirmed by passaging twice for 14 days postinoculation. GAKV particles were observed using a transmission electron microscope (Figure 2A). In addition, the number of infectious GAKV particles was 1 × 10^6^ PFU/mL, as quantified using the plaque assay (Figure 2B).

### 3.3. Nearly Whole-Genome Sequencing of Shrew-Borne Henipaviruses Using NGS

We performed host rRNA depletion on SISPA-based MiSeq of the kidney tissue of Cl17-46, resulting in two contigs (602 and 17,892 nt in length) with significant similarities in the genomic sequence of paramyxoviruses. The NGS of Cl17-46 generated 245,846 reads, and the number of viral genome sequences was 3608 (Table S5). To obtain whole-genome sequences of shrew-borne paramyxovirus, RNA-Seq (HiSeq) was performed on samples from the kidney tissues of Cl17-46 and Cs17-65 and on cells infected with the paramyxovirus isolated from Cl17-32. The genomic sequences of henipaviruses were identified across 23 contigs (408–18,382 nt in length) with significant similarities. The number of viral genome reads was 14,779,024, 6497, and 50,987 from Cl17-32, Cl17-46, and Cs17-65, respectively (Table S6). Nearly complete genome sequences of GAKV and DARV were obtained except for both 3′ and 5′ terminal ends. The genomic sequences of GAKV and DARV have been deposited in GenBank (accession number: MZ574407–MZ574409).

### 3.4. Genome Structure of GAKV and DARV

**Figure 3:**
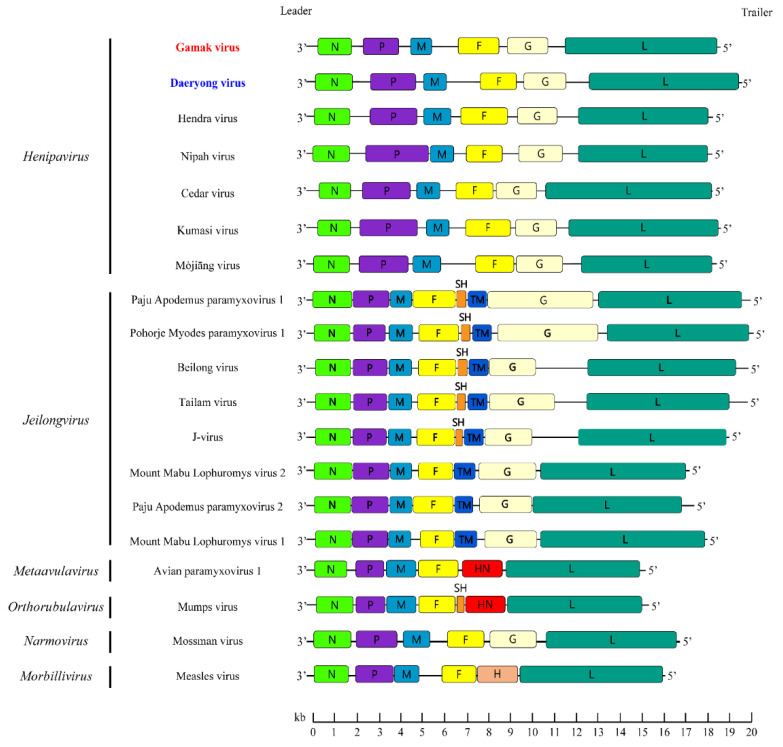
Organization of the genomes of the paramyxoviruses Gamak and Daeryong viruses, in individuals of *Crocidura*. The genomic configurations of related paramyxoviruses are shown. The genomes of paramyxoviruses comprise 6 to 8 coding regions: 3′ N-P-M-F-SH-TM-G, HN, or H-L 5′. The colored boxes represent coding regions for each gene: N, yellow-green; P, purple; M, sky blue; F, yellow; SH, orange; TM, blue; G, light yellow; HN, red; H, Chilean pink; L, viridian. A scale bar of the length is shown under the genome structure. This figure was constructed by using Adobe Illustrator 2021 (http://www.adobe.com/products/illustrator.html, 3 March 2021).

The genomes of GAKV and DARV revealed 18,460 nt and 19,471 nt in length, containing GC contents of 39.96–40.02% and 33.33%, respectively. GAKV and DARV presented a genomic organization consisting of the order 3′-N-P-M-F-G-L-5′ (Figure 3). The N, M, F, G, and L genes encode a single protein, whereas the P gene encodes multiple accessory proteins: the viral phosphoprotein, C protein, and V/W protein. GAKV and DARV possessed a putative RNA editing site (TTAAAAAAGGCA) of the P gene, a conserved motif sequence (YTAAAARRGGCA) in the members of the genus *Henipavirus*. The 3′ leader and 5′ trailer sequences were a length of 52 nt and 25 nt, respectively. Tables S7 and S8 show the start, stop, and intergenic region (IGR) sequences of GAKV and DARV in shrews.

### 3.5. Phylogenetic Analysis

**Figure 4:**
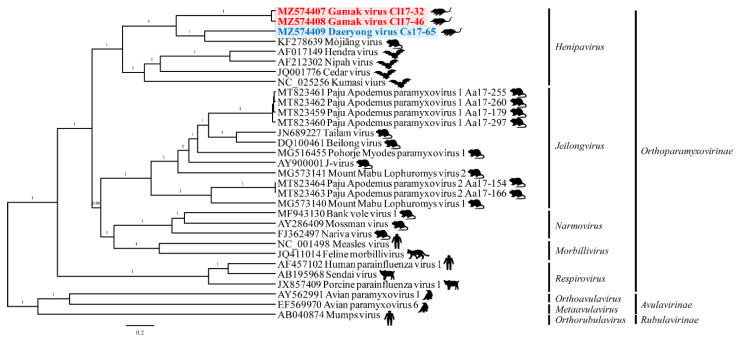
A phylogenetic tree of Gamak virus, Daeryong virus, and multiple paramyxoviruses using nearly whole-genome sequences. Phylogenetic inferences were conducted by BEAST (v1.10.4) with default priors and assuming homochromous tips. The Markov chain Monte Carlo analysis was run until sufficient sample sizes (ESS > 200) were acquired. The maximum clade credibility tree from the posterior tree distribution was summarized byTreeAnnotator (v2.5.4), using a 10% burn-in. *Paramyxoviridae* strains were utilized as reference sequences for this phylogenetic analysis. Red and blue bold texts indicate GAKV and DARV, respectively.

The phylogenetic analysis of all 26 currently recognized *Orthoparamyxovirus* species elucidated that GAKV and DARV belong to the genus *Henipavirus* and are closely related to the henipaviruses (Figure 4). In addition, the amino acid sequences (N, P, M, F, G, and L) of GAKV and DARV showed the phylogenetic shape comparable to the viral RNA genome sequences among major paramyxoviruses (Figure S1). The genetic cluster of GAKV and DARV shared a common ancestor with MojV with an amino acid, similarity to the L gene of 62.9% and 74.1%, respectively (Table S9). 

The partial L genomic sequences of GAKV and DARV strains (13,790–14,215 nt and 14,849–15,311 nt, respectively) belong to the genus *Henipavirus*, subfamily *Orthoparamyxovirinae* (Figure S2). Consistently, the phylogenies of GAKV and DARV were closely related to those of MojV, HeV, NiV, Cedar virus (CedV), and Kumasi virus (KV).

### 3.6. Co-Phylogeny of Paramyxovirus and Host

**Figure 5:**
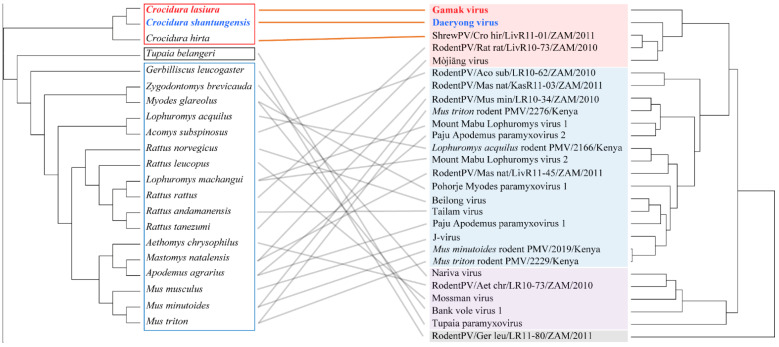
Tanglegram comparing the phylogenies between paramyxoviruses and their reservoir hosts (insectivores and rodents). The tanglegram was generated using the R package, using Bayesian consensus tree, based on the nucleotide sequences of the paramyxoviral *L* gene (right panel) and *cytochrome* *b* gene of mitochondrial DNA sequences of the host species (left panel). Letters for taxa are indicated in red for *Crocidura lasiura* in the left panel and Gamak virus in the right panel, respectively. Letters for taxa are shown in blue for *Crocidura shantungensis* in the left panel and Daeryong virus in the right panel, respectively. The left panel shows shrew species in the red box, rodent species in the blue box, and mole species in the black box. The right panel shows genus *Henipavirus* in the red box, genus *Jeilongvirus* in the blue box, genus *Narmovirus* in the purple box, and outgroup in the grey box.

Cophylogeny mapping demonstrated the segregation of paramyxoviruses according to the subfamily of their reservoir hosts, using consensus trees based on the L protein amino acid sequences (Figure 5). The phylogenetic positions of GAKV, DARV, and shrew paramyxovirus (Shrew PV) mirrored the phylogenetic relationships of the shrew species. In contrast, the phylogenetic positions of viruses from rodents such as Bank Vole virus (BaVV) and Pohorje Myodes paramyxovirus 1 (PMPV-1) from *Myodes glareolus*, Mount Mabu Lophuromys virus 1 (MMLV-1) and Mount Mabu Lophuromys virus 2 (MMLV-2) from *Lophuromys machangui*, and PAPV-1 and PAPV-2 from *Apodemus agrarius*, were not directly matched, as per the tanglegram between the virus and host species.

### 3.7. Characterization of Innate Immune Responses to GAKV in Human Lung Epithelial Cells

**Figure 6:**
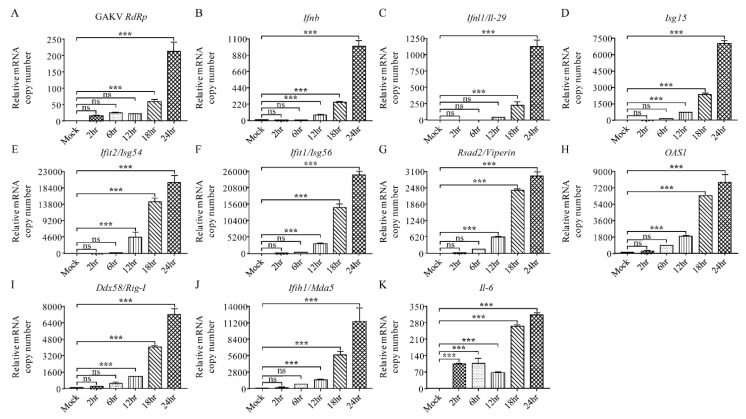
Enhanced expression of viral and innate antiviral genes in Gamak virus (GAKV)-infected A549 cells. A549 cells were infected with a GAKV at multiplicity of infection of 0.02. Total RNA was extracted and analyzed by quantitative reverse transcription-polymerase chain reaction (qRT-PCR) for the induction of (**A**) GAKV *RdRp*, (**B**) interferon-β (*Ifn-β*), (**C**) *Ifnl1/Il-29*, (**D**) interferon-stimulated gene product-15 (*ISG-15*), (**E**) *Ifit2/Isg54*, (**F**) *Ifit1/Isg56*, (**G**) radical S-adenosyl methionine domain containing 2 (*Rsad2/Viperin*), (**H**) 2′, 5′-oligoadenylate synthetase 1 (*OAS1*), (**I**) *Ddx58/Rig-I*, (**J**) *Ifih1/Mda5*, and (**K**) interleukin-6 (*Il-6*) at 2, 6, 12, 18, and 24 h postinfection. Error bars show the standard deviation of triplicate measurements in a representative experiment. *** *p* < 0.001, one-way analysis of variance (ANOVA) test; ns: non-significant.

To examine the infectivity and expression of innate antiviral genes in human lung epithelial cells, GAKV infected A549 cells for 2, 6, 12, 18, and 24 h. The replication of GAKV occurred and sequentially increased at 2, 6, 12, 18, and 24 hpi (Figure 6A). GAKV infection upregulated the mRNA copy numbers of type I/III IFN and ISGs, including *Ifn-β*, *Ifnl1/Il-29*, IFN-stimulated gene product 15 (*ISG-15*), *Ifit2/Isg54*, and *Ifit1/Isg56* at 12, 18, and 24 hpi (Figure 6B–F). The augmented induction of innate antiviral genes, radical S-adenosyl methionine domain containing 2 (*Rsad2/Viperin*), 2′, 5′-oligoadenylate synthetase 1 (*OAS1*), *Ddx58/Rig-I,* and *Ifih1/Mda5*, was observed at 12, 18, and 24 hpi (Figure 6G–J). The *Il-6* mRNA gene copies were rapidly increased at 2, 6, 12, 18, and 24 hpi (Figure 6K). In summary, these data demonstrated that GAKV infection elicited the induction of innate antiviral genes in human lung epithelial cells.

## 4. Discussion

We characterized two novel paramyxoviruses, GAKV and DARV, identified in *Crocidura* species, ROK. GAKV and DARV showed a high positive rate: GAKV was found in five regions, while DARV was detected in one region. The genomic structure (3′-N-P-M-F-G-L-5′) of these viruses is representative of the genus of henipaviruses, including HeV, NiV, MojV, CedV, and KV. The phylogenies of GAKV and DARV shaped independent genetic lineages corresponding to the paramyxovirus species distinctive criterion (the L gene amino acid distance of <0.82 for the *Orthoparamyxovirinae*) [26]. These observations suggest that GAKV and DARV are genetically distinct novel shrew-borne paramyxoviruses within the genus *Henipavirus* in the family *Paramyxoviridae*.

Shrews and rodents constitute the two largest groups of mammalian species [9]. They have been characterized as reservoir hosts of emerging zoonotic viruses that cause human diseases [54,55]. Recently, many studies have been conducted on animal viromes in wildlife [56,57,58]. Using viral metagenomics techniques, influenza virus and herpesvirus were discovered in house shrews, urban rats, and bats [58]. In addition, arenavirus, astrovirus, hantavirus, hepatitis E virus, and ZIKV were detected in shrews and rodents [33,55,58]. Recently, we discovered that striped field mice harbored PAPV-1 and -2 [30]. In addition, BaVV and PMPV-1 were found in the bank vole (*Myodes glareolus*), and MMLV-1 and MMLV-2 were carried by Rungwe brush-furred rats (*Lophuromys machangui*) [29,59]. In this study, we identified novel paramyxoviruses in samples from *C. lasiura* and *C. shantungensis* individuals. 

Cophylogenetic relationships of zoonotic viruses and natural reservoir hosts play a critical role in understanding the evolutionary process for coadaptation, spillover, and host sharing [60,61,62]. Multiple natural reservoirs harbor *Orthohantavirus* species, and in most cases, it is a specific relationship of “one virus–one host” [63]. The congruent relationships between whole groups of hantaviruses and (sub)families of hosts proposed a view of the long-term coevolution of these viruses with their hosts [64,65]. However, recent studies, based on cophylogenetic reconciliation and estimation of evolutionary rates and divergence times, demonstrate that local host-specific adaptation and preferential host switching account for the phylogenetic similarities between viruses and their mammalian hosts [66,67]. Our cophylogenetic analysis indicates that shrew-borne henipaviruses and their host species have coadapted together differently from the phylogenies of rodent-borne paramyxoviruses. Thus, continuous and large-scale investigations will provide insights into the evolutionary history and dynamics of paramyxoviruses, harbored by shrews and rodents, in the family *Orthoparamyxoviridae*.

Triggering of innate antiviral genes modulates viral infectious diseases in humans and animals [68,69]. The expression of antiviral genes, *Mx1*, *Rsad2/Viperin*, *Isg15*, and *OAS1*, was highly induced during early NiV and HeV infections in ferrets [70]. These robust innate antiviral responses were insufficient to prevent viral dissemination and tissue damage in vivo [71]. The isolate of GAKV from *C. lasiura* was evaluated for the innate antiviral response in human lung epithelial cells. We found that GAKV enabled replication and elicited the rapid induction of type I/III IFNs, ISGs, and pro-inflammatory cytokines in A549 cells. These results demonstrated that the novel paramyxovirus may infect and induce pro-inflammatory responses via the human respiratory tract. The characterization and effect of innate antiviral responses to shrew-borne henipaviruses in humans await for further studies.

In conclusion, this study reports two generically distinct novel paramyxoviruses, GAKV in *C. lasiura* and DARV in *C. shantungensis*. Phylogenetic inference and genomic characterization demonstrated that these viruses belong to henipaviruses within the family *Paramyxoviridae*. Infection with GAKV showed the upregulation of multiple human innate antiviral genes in vitro. Therefore, these results facilitate understanding of the molecular epidemiology, genetic characteristics and diversity, and zoonotic potential of shrew-borne paramyxoviruses, ROK. These observations raise awareness and caution among virologists and physicians about the henipa-like paramyxoviruses in shrews.

## Figures and Tables

**Table 1:** Prevalence of paramyxovirus infections in *Crocidura* species individuals captured during 2017–2018, Republic of Korea.

Year	Species	Province	Location	Number of Samples	Positivity for Paramyxovirus (%)
2017	*Crocidura* *lasiura*	Gangwon	Cheorwon	4	2/4 (50.0)
			Chuncheon	17	5/17 (29.4)
			Hwacheon	5	0/5
		Gyeonggi	Paju	11	6/11 (54.5)
			Yeoncheon	20	3/20 (15.0)
		Gyeongsangnam	Changnyeong	2	1/2 (50.0)
	*Crocidura* *shantungensis*	Gangwon	Chuncheon	8	5/8 (62.5)
		Gyeonggi	Paju	2	0/2
			Yeoncheon	2	0/2
	Subtotal			71	22/71 (31.0)
2018	*Crocidura* *lasiura*	Gangwon	Chuncheon	10	3/10 (30.0)
			Hongcheon	2	0/2
			Pyeongchang	2	0/2
		Gyeonggi	Osan	2	0/2
			Paju	7	0/7
			Pocheon	4	0/4
			Suwon	2	0/2
			Yeoncheon	6	0/6
	*Crocidura* *shantungensis*	Gangwon	Chuncheon	1	1/1 (100)
			Pyeongchang	1	0/1
		Gyeonggi	Paju	4	0/4
			Pocheon	2	0/2
			Yeoncheon	1	0/1
	Subtotal			44	4/44 (9.1)
		**Total**		**115**	**26/115 (22.6)**

## Data Availability

All the data generated for this publication have been included in the current manuscript.

## Supplementary Materials

The following are available online at https://www.mdpi.com/article/10.3390/v13102020/s1, Figure S1: Phylogenetic trees using the coding sequences (CDSs) of *N, P, M, F, G*, and *L* genes of Gamak virus (GAKV) and Daeryong virus (DARV); Figure S2: Phylogenetic analyses of the paramyxoviruses using partial genome sequences of L gene; Table S1: Sequences of primers for quantitative reverse transcription-polymerase chain reaction; Table S2: Characteristics of *Crocidura* species infected with Gamak virus (GAKV) and Daeryong virus (DARV) and the nucleotide position of GAKV and DARV RNA obtained in this study; Table S3: Molecular prevalence of Gamak virus (GAKV) by region, sex, weight, and season in *C. lasiura*, the Republic of Korea from 2017 to 2018; Table S4: Molecular prevalence of Daeryong virus (DARV) by region, sex, weight, and season in *C. shantungensis*, the Republic of Korea from 2017 to 2018; Table S5: Genome coverage of consensus sequences for Gamak virus using next-generation sequencing (SISPA-based MiSeq) method; Table S6: Genome coverage of consensus sequences for Gamak and Daeryong viruses using next-generation sequencing (RNA-Seq based HiSeq) method; Table S7: Sequences of intergenic regions (IGRs) and transcriptional stop and start signals of Gamak virus; Table S8: Sequences of intergenic regions (IGRs) and transcriptional stop and start signals of Daeryong virus; Table S9: Amino acid similarities of GAKV and DARV with other viruses in *P**aramyxoviridae.*
