# Supplementary material for: Discovery and Genetic Characterization of Novel Paramyxoviruses Related to the Genus Henipavirus in Crocidura Species in the Republic of Korea
Source: Viruses. 2021 Oct 7;13(10):2020. doi: 10.3390/v13102020 (PMC8537881; doi:10.3390/v13102020)
Supplement: Supplementary file 1 [file Supplementary_Material_Rebuttal_submission.pdf › Supplementary_Material_Rebuttal_submission.pdf]

Supplementary Material

Supplementary Figures

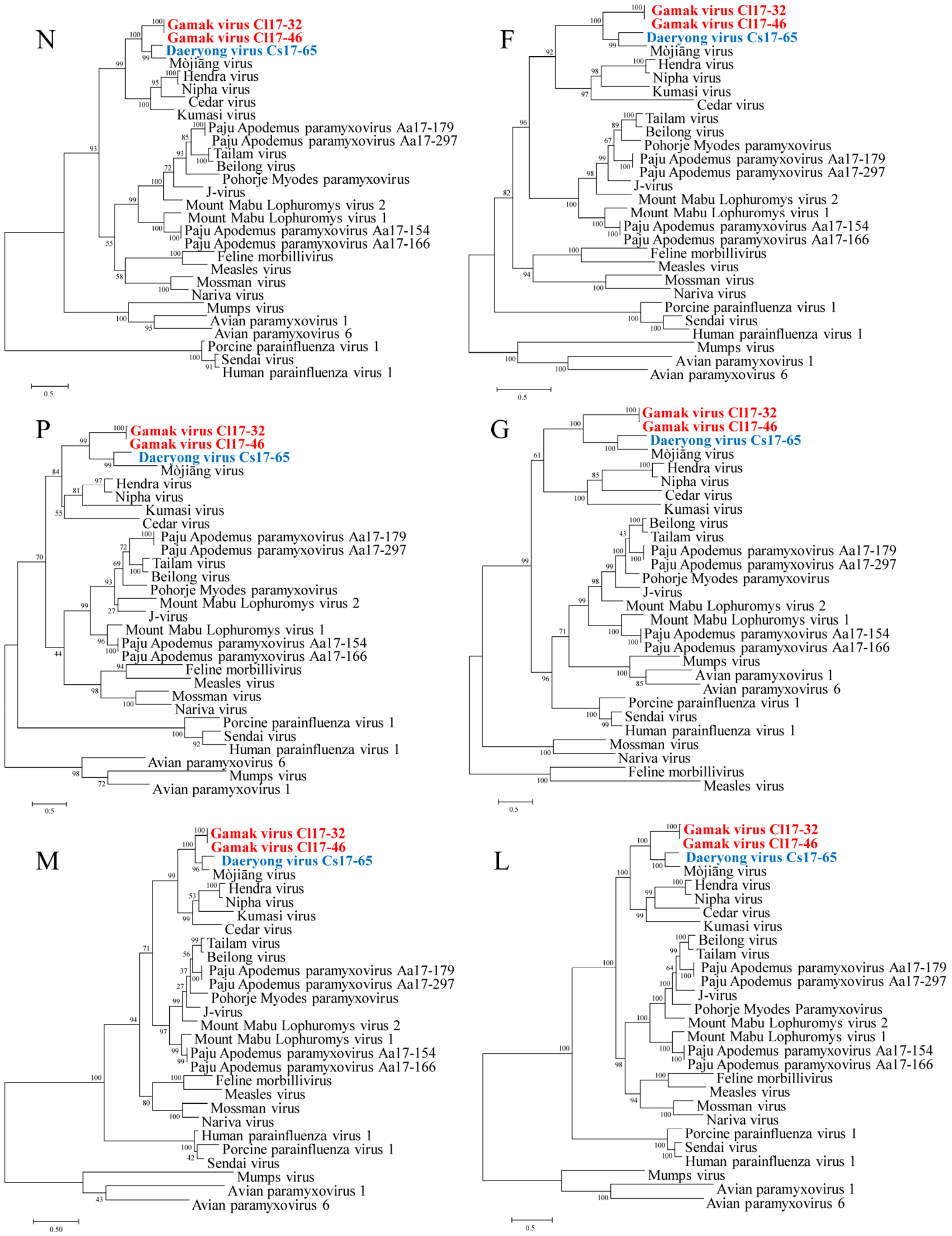

Figure S1. Phylogenetic trees using the coding sequences (CDSs) of *N, P, M, F, G*, and *L* genes of Gamak virus (GAKV) and Daeryong virus (DARV). The phylogenetic trees of orthoparamyxoviruses were generated using the maximum likelihood method with distribution models based on the N, P, M, F, G, and L proteins. Topologies were evaluated using a bootstrap analysis of 1,000 iterations. Multiple *Paramyxoviridae* strains were implemented as reference sequences for this phylogenetic inference. Red and blue bold texts indicate GAKV and DARV, respectively.

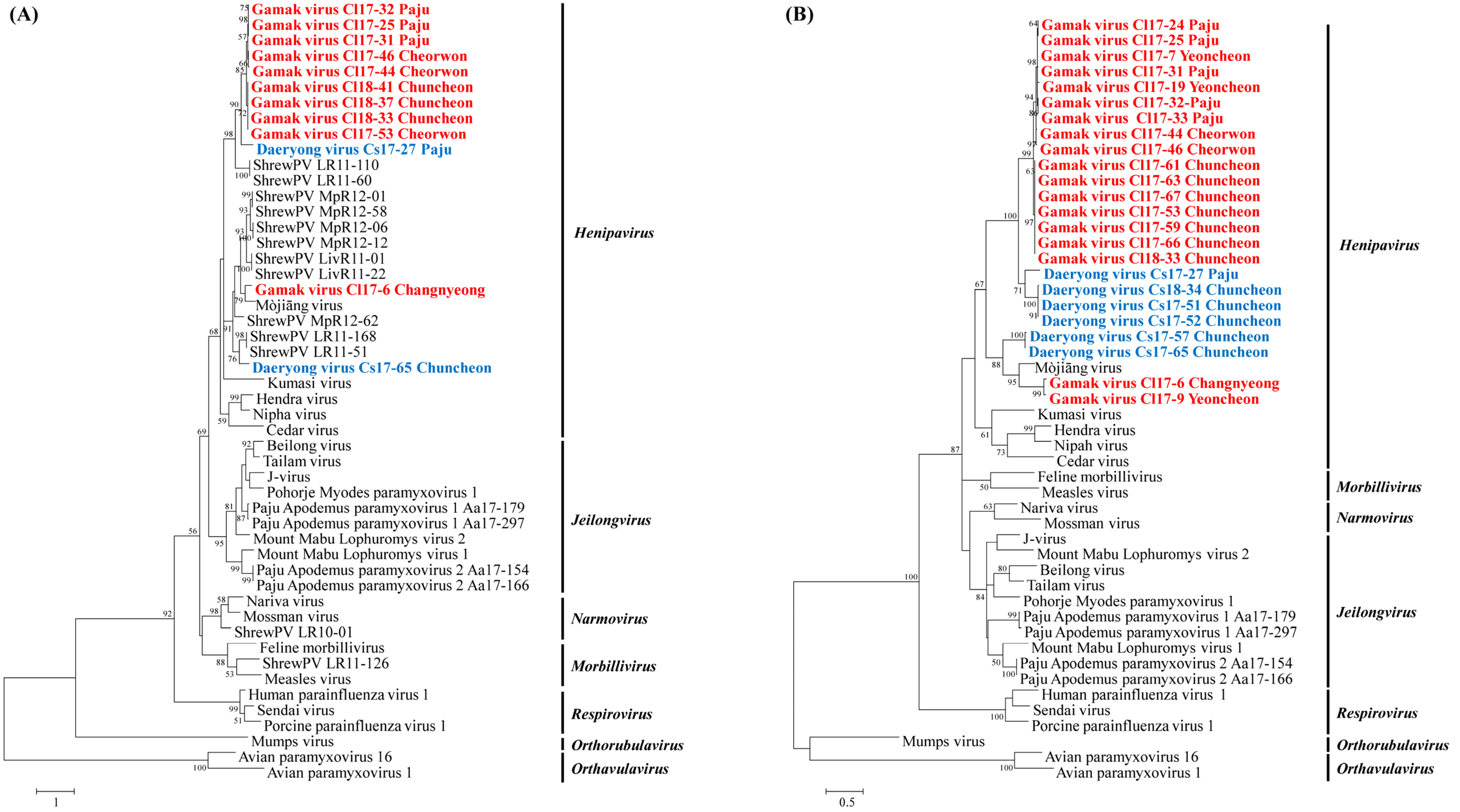

Figure S2. Phylogenetic analyses of the paramyxoviruses using partial genome sequences of L gene. Phylogenetic trees were generated using the maximum likelihood method via alignment of the rodent- and shrew-borne paramyxoviruses with other members of the *Paramyxovirinae* subfamily. Phylogenetic analysis of (A) 13,790–14,215 nt (426 bp) and (B) 14,849–15,311 nt (463 bp) of the *L* gene from *Henipavirus* with the corresponding region from other members of the *Paramyxovirinae* subfamily. Support for the topologies was assessed via bootstrapping for 1,000 iterations.

Supplementary Tables

Table S1. Sequences of primers for quantitative reverse transcription-polymerase chain reaction.

Genes	Forward (Sense, 5′–3′)	Reverse (Antisense, 5′–3′)
Human b-actin	AGAGCTACGAGCTGCCTGAC	CGTGGATGCCACAGGACT
Human GAPDH	GCAAATTCCATGGCACCGT	TCGCCCCACTTGATTTTGG
Ifnβ	GTCAGAGTGGAAATCCTAAG	ACAGCATCTGCTGGTTGAAG
Il-29	CGCCTTGGAAGAGTCACTCA	GAAGCCTCAGGTCCCAATTC
Isg15	GCGAACTCATCTTTGCCAGTA	CCAGCATCTTCACCGTCAG
Ifit2/Isg54	ATCCCCCATCGCTTATCTCT	CCACCTCAATTAATCAGGCACT
Ifit1/Isg56	GGATTCTGTACAATACACTAGAAACCA	CTTTTGGTTACTTTTCCCCTATCC
Rsad2/Viperin	TGCTTTTGCTTAAGGAAGCTG	TCTACTTTGCAGAACCTCACCA
OAS-1	GAAGGCAGCTCACGAAACC	AGGCCTCAGCCTCTTGTG
Ddx58/Rig-I	CAGACAGATCCGAGACACTA	TGCAAGACCTTTGGCCAGTT
Ifih1/Mda5	GAGGAATCAGCACGAGGAATAA	TCAGATGGTGGGCTTTGAC
Il-6	GCCCAGCTATGAACTCCTTCT	GCGGCTACATCTTTGGAATCT
GAKV *RdRp*	CAGTGCAGAACGAGCTCAAGCATA	TGTGTACTTATTATGGTTTCGGTTGC

Table S2. Characteristics of *Crocidura* species infected with Gamak virus (GAKV) and Daeryong virus (DARV) and the nucleotide position of GAKV and DARV RNA obtained in this study.

Type of Paramyxovirus	Sample	Site (City/Province)	Trapping date	Gender	Weight (g)	Vital status	Nucleotide (nt) position of Paramyxovirus RNA
GAKV	Cl17-32 [a,b]	Paju/Gyeonggi	Oct. 27, 2017	Male	8.0	Alive	1–18,460
	Cl17-46 [a]	Cheorwon/Gangwon	Nov. 21, 2017	Male	8.0	Dead	1–18,460
	Cl17-6	Changnyeong/Gyeongsangnam	Jun. 29, 2017	Female	12.5	Dead	13,790–14,215; 14,849-15,311
	Cl17-7	Yeoncheon/Gyeonggi	Sep. 28, 2017	Male	8.0	Alive	14,849-15,311
	Cl17-9	Yeoncheon/Gyeonggi	Sep. 28, 2017	Male	11.0	Dead	14,849-15,311
	Cl17-19	Yeoncheon/Gyeonggi	Sep. 29, 2017	Male	10.0	Dead	14,849-15,311
	Cl17-24	Paju/Gyeonggi	Oct. 19, 2017	Male	9.5	Dead	14,849-15,311
	Cl17-25	Paju/Gyeonggi	Oct. 19, 2017	Male	11.5	Dead	13,790-14,215; 14,849-15,311
	Cl17-27	Paju/Gyeonggi	Oct. 19, 2017	Female	5.5	Dead	13,790–14,215; 14,849-15,311
	Cl17-31	Paju/Gyeonggi	Oct. 27, 2017	Male	8.5	Dead	13,790-14,215; 14,849-15,311
	Cl17-33	Paju/Gyeonggi	Oct. 27, 2017	Male	9.0	Alive	13,790-14,215; 14,849-15,311
	Cl17-44	Cheorwon/Gangwon	Apr. 6, 2017	Male	11.0	Dead	13,790-14,215; 14,849-15,311
	Cl17-59	Chuncheon/Gangwon	Oct. 29, 2017	Female	9.5	Dead	14,849-15,311
	Cl17-61	Chuncheon/Gangwon	Oct. 30, 2017	Male	10.2	Dead	14,849-15,311

	Cl17-63	Chuncheon/Gangwon	Oct. 29, 2017	Female	8.5	Dead	14,849-15,311
	Cl17-66	Chuncheon/Gangwon	Oct. 31, 2017	Female	11.4	Dead	14,849-15,311
	Cl17-67	Chuncheon/Gangwon	Nov. 8, 2017	Male	12.4	Dead	14,849-15,311
	Cl18-33	Chuncheon/Gangwon	Nov. 13, 2018	Male	10.5	Dead	13,790-14,215; 14,849-15,311
	Cl18-37	Chuncheon/Gangwon	Nov. 13, 2018	Male	7.0	Dead	13,790-14,215; 14,849-15,311
	Cl18-41	Chuncheon/Gangwon	Nov. 14, 2018	Male	7.5	Alive	13,790-14,215
DARV	Cs17-65 [a]	Chuncheon/Gangwon	Oct. 31, 2017	Female	5.1	Dead	1–19,475
	Cs17-51	Chuncheon/Gangwon	Sep. 28, 2017	Male	36.5	Dead	14,849-15,311
	Cs17-52	Chuncheon/Gangwon	Oct. 11, 2016	Female	44.5	Dead	14,849-15,311
	Cs17-53	Chuncheon/Gangwon	Apr. 18, 2017	Male	26.0	Dead	13,790-14,215
	Cs17-57	Chuncheon/Gangwon	Apr. 18, 2017	Female	28.0	Dead	14,849-15,311
	Cs18-34	Chuncheon/Gangwon	Oct. 16, 2018	Female	32.0	Dead	14,849-15,311

[a]: Whole genome sequences were completely obtained.
[b]: Cell culture-based isolates.

Table S3. Molecular prevalence of Gamak virus (GAKV) by region, sex, weight, and season in *C. lasiura*, the Republic of Korea from 2017 to 2018.

Category	Number of *C. lasiura*	RNA positivity of GAKV (%)*
**Region (n=94)**		
Gangwon	40	11/40 (27.5%)
Gyeonggi	52	9/52(17.3%)
Gyeongsangnam	2	1/2 (50.0%)
**Sex (n=94)**		
Males	58	16/58 (27.6%)
Females	36	5/36 (13.9%)
**Weight (n=94)**		
<10g (Subadults)	54	11/54 (20.4%)
10–20g (Adults)	40	10/40 (25.0%)
**Season (n=94)**		
Spring (Mar.–May)	9	1/9 (11.1%)
Summer (Jun.–Aug.)	2	1/2 (50.0%)
Autumn (Sep.–Nov.)	79	19/79 (24.1%)
Winter (Dec.–Feb.)	4	0/4
**Total**	**94**	**21/94 (22.3%)**

*The positive rate of GAKV RNA indicates the detection of the partial L gene (targeting pan-*Orthoparamyxovirinae* and the genera *Respirovirus*, *Morbillivirus*, and *Henipavirus*) using RT-PCR and Sanger sequencing.

Table S4. Molecular prevalence of Daeryong virus (DARV) by region, sex, weight, and season in *C. shantungensis*, the Republic of Korea from 2017 to 2018.

Category	Number of *C. shatungensis*	RNA positivity of DARV (%)*
**Region (n=21)**		
Gangwon	10	5/10 (50.0%)
Gyeonggi	11	0/11
Gyeongsangnam	0	0/0
**Sex (n=21)**		
Males	9	1/9 (11.1%)
Females	12	4/12 (33.3%)
**Weight (n=21)**		
<5g (Subadults)	14	3/14 (21.4%)
5–9g (Adults)	5	2/7 (28.6%)
**Season (n=21)**		
Spring (Mar.–May)	2	0/2
Summer (Jun.–Aug.)	0	0/0
Autumn (Sep.–Nov.)	19	5/19 (26.3%)
Winter (Dec.–Feb.)	0	0/0
**Total**	**21**	**5/21 (23.8%)**

*The positive rate of DARV RNA indicates the detection of the partial L gene (targeting pan-*Orthoparamyxovirinae* and the genera *Respirovirus*, *Morbillivirus*, and *Henipavirus*) using RT-PCR and Sanger sequencing.

Table S5. Genome coverage of consensus sequences for Gamak virus using next-generation sequencing (SISPA-based MiSeq) method.

NGS method	Origin	Strains	Total reads	Read mapped to reference/Total reads (%)	Crocidura paramyxoviruses	
					Read mapped to reference	Depth of coverage*
SISPA-based MiSeq	Kidney	Cl17-46[a]	245,846	1.468	3,608	28.703

*Depth of coverage was calculated using the number of mapped reads [(average read length × number of reads that matched the reference)/(reference genome size)].

[a]: Gamak virus 18,460 nt

SISPA: sequence-independent, single-primer amplification

Table S6. Genome coverage of consensus sequences for Gamak and Daeryong viruses using next-generation sequencing (RNA-Seq based HiSeq) method.

NGS method	Origin	Strains	Total reads	Read mapped to reference/Total reads (%)	Crocidura paramyxoviruses	
					Read mapped to reference	Depth of coverage*
RNA-Seq based HiSeq	Vp	Cl17-32[a]	83,500,574	17.699	14,779,024	80665.238
	Kidney	Cl17-46[a]	72,742,132	0.009	6,497	35.356
		Cs17-65[b]	83,682,946	0.061	50,987	278.016
**Total**			**239,925,652**	**6.184**	**14,836,508**	**80,978.61**

*Depth of coverage was calculated using the number of mapped reads [(read length × number of reads that matched the reference)/(reference genome size)].

Vp: Cell culture-based isolates

[a]: Gamak virus 18,460 nt

[b]: Daeryong virus 19,475 nt

Table S7. Sequences of intergenic regions (IGRs) and transcriptional stop and start signals of Gamak virus.

Genes	Gene stop	IGR	Gene Start
/N		CTT	AAGAATCAAA
N/P	TTAAGAAAAA	CTT	AAGAATCAAA
P/M	TTAAGAAAAA	CTT	AAGAGTCAAA
M/F	TTAGAAAAAA	CTT	AAGAATCAAA
F/G	TTAAGAAAAA	CTT	AAGAATCAAA
G/L	TTAAGAAAAA	CTT	AAGAATCAAA
L/	TTAAGAAAAA	CTT	
Consensus sequences	TTARRAAAAA	CTT	AAGARTCAAA

Table S8. Sequences of intergenic regions (IGRs) and transcriptional stop and start signals of Daeryong virus.

Genes	Gene stop	IGR	Gene Start
/N		CTT	AGGACTCAGG
N/P	TTATAAAAAA	CTT	AGGATGCAAG
P/M	TTAAGAAAAA	CTT	AGGGGTAAAG
M/F	TTAAAGAAAA	CTT	AGGACGTCAA
F/G	TTATAAAAAA	CTT	AGGGGTCAAG
G/L	TTAAGAAAAA	CTT	AGGTGCAATG
L/	ATATAAAAAA	CTT	
Consensus sequences	WTAWRRAAAA	CTT	AGGDBBHMDR

Table S9. Amino acid similarities of GAKV and DARV with other viruses in *Paramyxoviridae*.

Genus	Virus	GAKV						DARV					
		N	P	M	F	G	L	N	P	M	F	G	L
Henipavirus	**GAKV**	98.7	96.3	99.4	99.1	99.4	98.5	57.9-60.1	30.7-31.3	76.8	57.5-57.7	28.8-28.9	62.9
	**DARV**	57.9-60.1	30.7-31.3	76.8	57.5-57.7	28.8-28.9	62.9	-	-	-	-	-	-
	HeV	47.0-48.1	22.7-23.0	60.1-60.4	39.7	17.4	52.0-52.1	46.4	22.0	59.5	39.0	18.4	51.9
	NiV	47.7-48.7	23.9-24.2	59.5-59.8	40.3	17.8	52.4	46.6	18.7	59.3	39.9	18.3	52.2
	MojV	58.4-58.6	27.4-29.0	80.3-80.6	56.9-57.1	29.9	62.3-62.4	69.6	36.0	83.1	67.3	49.3	74.1
	CedV	45.7	19.1-19.4	57.5	33.4	16.9	49.4-49.5	45.7	19.0	57.8	34.1	17.3	49.8
	KV	46.1	25.6-25.7	56.9	38.3	16.3	49.0-49.3	46.1	19.3	58.9	39.2	18.3	49.2
Jeilongvirus	PAPV-1	34.7-35.4	18.3	55.3	37.9-38.4	18.6	49.6	36.4	20.4-21.4	53.5	36.8-37.0	19.9	49.0-49.2
	PAPV-2	33.3-34.6	21.4-22.9	57.2-57.4	35.5-35.7	17.1-17.3	49.7	34.9-35.8	20.0	56.0-56.2	35.8-35.9	17.8	50.7-50.8
	TaiV	34.7-34.9	19.4-19.6	53.5	37.5-37.7	18.4	48.7-48.8	36.6	17.3	53.2	35.5	18.6	49.5
	BeiV	34.7-35.4	18.5-19.4	55.0	38.4-38.6	18.6	48.7-48.8	36.2	14.3	54.1	35.3	19.2	49.0
	JV	33.7	18.6-18.8	52.9	37.3-37.5	17.9	48.6-48.8	36.0	20.4	52.6	36.2	20.5	48.7
	MMLV-1	35.6-36.0	22.1-22.5	56.0	34.8-35.0	20.3-20.6	49.1-49.3	35.3	18.4	57.5	36.8	18.4	49.9
	MMLV-2	33.9	20.9-22.5	57.1-57.4	38.3	19.7	50.3-50.4	36.2	19.5	53.8	37.2	17.8	50.5
	PMPV-1	34.8-35.0	21.6-22.8	54.7	36.0	19.7	49.2-49.3	35.0	18.5	52.9	35.1	19.1	50.5
Morbillivirsu	FeMV	31.8	17.7-17.9	45.4	32.2	12.4	45.2-45.3	32.2	18.5	46.0	32.8	9.7	45.7
	MV	31.2-31.6	16.8-17.4	46.0	31.8	12.3	46.9-47.1	32.4	18.1	46.6	32.1	10.9	46.7
Narmovirus	MossV	34.8-35.2	12.7-14.6	48.2	29.9	13.8	47.4-47.5	36.2	19.2	48.2	32.6	13.5	48.1
	NarV	36.2-36.4	14.7-15.7	49.4	29.0	14.0-14.2	46.3-46.4	36.4	16.7	49.1	30.6	12.9	46.3
Respirovirus	PPIV-1	20.0	11.1-11.5	34.3	25.9	19.1	37.5-37.9	18.3	7.8	34.7	26.9	18.6	39.6
	SenV	19.5	12.0-12.3	34.6	26.4-26.5	18.4-18.6	36.7-37.4	20.6	9.7	34.4	27.5	19.8	38.6
	HPIV-1	19.5-19.8	11.4-12.7	35.8	25.8-25.9	18.3	36.8-37.3	20.0	13.6	33.5	26.2	18.1	39.5
Orthorubulavirus	MuV	24.5	9.4-12.1	17.9	25.8-25.9	13.4	27.2-27.3	25.7	10.3	17.6	25.1	13.1	26.3
Orthoavulavirus	APMV-1	27.4-27.6	17.5	19.6	24.6-24.8	15.3	24.9	25.3	17.7	19.4	26.9	13.5	26.0
Metaavulavirus	APMV-6	26.7-27.1	13.0-14.0	22.3	25.2	15.5	25.8-26.8	27.5	10.2	22.5	28.2	15.2	26.7

Similarities are indicated per protein and are expressed as percentages. GAKV, Gamak virus; DARV, Daeryong virus; HeV, Hendra virus; NiV, Nipah virus; MojV, Mòjiāng virus; CedV, Cedar virus; KV, Kumasi virus; PAPV-1, Paju Apodemus paramyxovirus 1; PAPV-2, Paju Apodemus paramyxovirus 2; TaiV, Tailam virus; BeiV, Beilong virus; JV, J-virus; MMLV-1, Mount Mabu Lophuromys virus 1; MMLV-2, Mount Mabu Lophuromys virus 2; PMPV-1, Pohorje Myodes paramyxovirus 1; FeMV, Feline morbillivirus; MeV, Measles virus; PPIV-1, Porcine parainfluenza virus 1; SenV, Sendai virus; HPIV-1, Human parainfluenza virus 1; MuV, Mumps virus; APMV-1, Avian paramyxovirus 1; APMV-6, Avian paramyxovirus 6.
